# Use of Peripheral Nerve Blocks for Total hip Arthroplasty

**DOI:** 10.1007/s11916-024-01287-7

**Published:** 2024-06-22

**Authors:** Leon Grinman, Brett Elmore, Alberto E. Ardon, Adnan Hussain, Mohammed Faysal Malik, Nadia Hernandez, Mackenzie Janice Jacoby

**Affiliations:** 1 Department of Anesthesiology and Perioperative Medicine, University of Virginia, PO Box 800710, Charlottesville, VA 22908-0710 USA; 2 Department of Anesthesiology and Perioperative Medicine, Mayo Clinic, Jacksonville, India; 3 Department of Anesthesiology and Perioperative Medicine, Henry Ford Health, Detroit, USA; 4 Department of Anesthesiology and Perioperative Medicine, University of Texas McGovern Medical School, Austin, USA

**Keywords:** Total hip arthroplasty, Regional anesthesia, Nerve block, Post-operative pain, Perioperative outcomes

## Abstract

**Purpose of Review:**

The purpose of this review is to summarize the recent literature regarding regional anesthesia (RA) techniques and outcomes for total hip arthroplasty (THA) in the face of changing surgical techniques and perioperative considerations.

**Recent Findings:**

Based on large meta-analyses, peripheral nerve blocks are indicated for THA. Each block has its own risks and benefits and data for outcomes for particular techniques are limited.

**Summary:**

New surgical techniques, improved use of multimodal analgesia, and improved ultrasound guided regional anesthetics lead to better pain control for patients undergoing THA with less associated risks. Block selection continues to be influenced by provider comfort, surgical approach, patient anatomy, and postoperative goals. Head-to-head studies of particular nerve blocks are warranted.

## Introduction

Total hip arthroplasty (THA), in which diseased native hip architecture is replaced by prosthetic components, is one of the most common surgical procedures performed in the United States [1]. The procedure has several indications, such as arthritis and trauma., While many times uncomplicated, THA can be associated with significant postoperative pain [2]. The use of regional anesthesia (RA) to help control postoperative pain has been shown to be important for patient recovery [3]. Several peripheral nerve block (PNB) techniques have been utilized in THA for postoperative analgesia. The lack of consensus on any one PNB for THA is due in part to the variety of surgical approaches as well as the complexity of hip innervation.

A recent meta-analysis of orthopedic literature describes the most common surgical approaches: posterior, lateral, and direct anterior [4]. The posterior approach involves making a large incision into the gluteus maximus to access the hip joint posteriorly. This ensures excellent exposure to the acetabulum and avoids disrupting the abductors, but places the sciatic nerve at risk due to its anatomical proximity. The lateral approach involves a smaller incision into the gluteus medius and minimus, therefore placing the superior gluteal nerve and artery at risk. As both approaches require splitting musculature to access the joint, there is increased trauma and inflammation to those structures [5].

In contrast, the director anterior approach (DAA) is unique for utilizing the intermuscular plane between sartorius and tensor fascia lata (TFL). This approach boasts a shorter recovery time, however, the risk of nerve damage, namely to the lateral femoral cutaneous nerve (LFCN) is higher, as well as an increased rate of periprosthetic fractures and a higher learning curve. Notably, the direct anterior approach is associated with less postoperative pain, as evidenced in 8 of the 11 RCTs analyzed in the meta-analysis, presumably due to less surgical trauma to the soft tissues [4]. Ultimately, there is ongoing debate regarding the optimal approach, and surgeon preference plays a large role in surgical decision making.

Each surgical approach attempts to provide access to the hip capsule while avoiding damage to its complex innervation. The anterior portion of the capsule is innervated by branches of the femoral, obturator and accessory obturator nerves, while the posterior capsule is innervated by branches of the sciatic nerve [6, [7]. This leads to a host of decisions that need to be made when considering a regional anesthetic plan. An ideal block would cover the bony aspect of the hip surgery, the capsular innervation, and the soft tissue transgressed for any given approach. Further, it should not interfere with the surgical approach and needs to avoid the potential for clinically significant motor dysfunction postoperatively. No single approach meets all of these criteria.

There are many PNBs described in recent literature to adequately provide analgesia to the hip. In this review, we will consider literature from the last five years assessing the efficacy of PNBs for THA, with special attention to the functional outcomes of patients receiving these interventions.

## Methods

A search was conducted by the lead author on PubMED/MEDLINE in March of 2024. Using the Advanced search function, Medical Subject Heading ([MeSH]) terms for Total Hip Arthroplasty [MeSH] were sequentially combined with the following regional techniques using [MeSH]: Paravertebral Block, Erector Spinae Plane Block, Lumbar Plexus Block, Quadratus Lumborum Block, Pericapsular Nerve Group Block, Lateral Femoral Cutaneous Nerve Block, Femoral Nerve Block, and Fascia Iliaca Compartment Block. In addition, Total Hip Arthroplasty [MeSH] was combined with Regional Nerve Block [MeSH]. See Table 1. These lists were combined in PubMED’s clipboard, which automatically removed duplicates. This combined list yielded 253 articles which were sent to the co-authors. Each co-author, based on their institutional practice and block familiarity, chose a particular block to review, and chose which articles to include in the final manuscript. Occasionally an article outside of the date scope of this list was included for context.

**Table 1:** Table 1

Items	Specification
Date of Search	March 21st 2024
Database	PubMED/Medline
***Search Terms used***	***Total Hip Arthroplasty [MeSH] AND Lumbar Plexus Block [MeSH] ; Total Hip Arthroplasty [MeSH] AND Paravertebral Block [MeSH] ; Total Hip Arthroplasty [MeSH] AND Erector Spinae Block [MeSH] ; Total Hip Arthroplasty [MeSH] AND Quadratus Lumborum Block [MeSH] ; Total Hip Arthroplasty [MeSH] AND Pericapsular Nerve Group [MeSH] ; Total Hip Arthroplasty [MeSH] AND Femoral Nerve Block [MeSH] ; Total Hip Arthroplasty [MeSH] AND Fascia Iliaca Block [MeSH] ; Total Hip Arthroplasty [MeSH] AND Lateral Femoral Cutaneous Nerve [MeSH] ; Total Hip Arthroplasty [MeSH] AND Peripheral Nerve Block [MeSH]***
***Inclusion and Exclusions***	***Within last five years (2019–2024); English language only***
***Selection process***	***Lists combined and duplicates removed via Pubmed clipboard function yielding 253; disseminated to authors for selection for subsequent block sections of manuscript.***

### Peripheral Nerve Blocks

We review here the most common approaches to anesthetizing the hip. Starting from the most proximal, we consider the paravertebral block (PVB), the erector spinae plan block (ESP), the lumbar plexus block (LPB), the quadratus lumborum block (QL), the pericapsular nerve group block (PENG), the fascia iliaca compartment block (FICB), and finally the femoral nerve and lateral femoral cutaneous blocks (FNB / LFCN).

### Paravertebral and Erector Spinae Plane Blocks

Recent studies assessing the efficacy of PVB for analgesia after THA are sparse, and most of these studies involve either small population sizes or retrospective analyses. Contextually, the lack of studies may be influenced by papers in the early 2000s suggesting that performance of PVB did not dramatically affect postoperative recovery. For example, in 2002 Bogoch et al. conducted a randomized controlled trial among a total of 115 patients that did not report any significant differences in either pain scores or opioid use when PVB was compared to sham block [8]. In one of the more recent studies addressing this topic, 30 patients received a PVB catheter at L3 and were compared to 30 patients who received an ESP catheter at L2 for post-THA analgesia [9]. No differences in opioid use were reported. The utility of PVB for THA may also be influenced by surgical technique. Both periarticular local anesthetic injection and anterior approach THA are rapidly becoming the de facto standard of care. Both mechanisms decrease postoperative pain compared to no periarticular injection or posterior-approach THA, respectively. Thus, the argument for deep peripheral nerve blocks such as PVB has steadily weakened in the past decade. For example, while one case series discussed the use of PVB for anterior approach THA, the authors did not compare analgesic or functional outcomes to either a historical control group or comparative literature [10]. At the same institution, from the personal experience of this author, the performance of PVB within the context of a multimodal analgesia protocol aimed at facilitating fast recovery and same-day discharge does not seem to significantly influence functional outcomes or the rate of successful discharge on the day of surgery.

Similarly, recent literature on the impact of ESP blocks on analgesia after THA is limited. One of the few RCTs comparing ESP to placebo by Lennon et al., reported no difference in pain scores or time to first ambulation [11]. Another RCT found no significant differences in pain scores or opioid use when comparing patients who received an L1 ESP to those who did not receive a block [12]. Similar to the limitations found in the PVB literature, studies assessing the impact of ESP may be influenced by practice patterns. For example, all patients in the Lennon trial received spinal blockade, oral multimodal analgesia, and periarticular injections, which is reflective of current practice and could negate the need for peripheral nerve blockade. Overall, current literature does not suggest that either paravertebral or erector spinae blocks are useful techniques for analgesia after hip arthroplasty.

### Lumbar Plexus Block

The lumbar plexus block (LPB) for lower limb surgery was initially described in the 1970s and gained popularity as an alternative to neuraxial analgesia following THA in the 2000s [13]. Multiple recent RCTs suggest that LPB has advantages when compared to neuraxial anesthesia. Sharma et al. found that the LPB group had increased time to first PCA bolus using continuous catheters, decreased pain on movement, and higher patient satisfaction compared to neuraxial group [14]. Zhang et al. found that LPB combined with sciatic nerve block resulted in reduced VAS scores, higher MOCA scores at 12 days, and lower inflammatory cytokines at 10 days compared to CSE [15]. Kacmaz et al. found LPB combined with sciatic nerve block had similar outcomes to spinal anesthesia in opioid use for 48 h, 30-day mortality, ICU admission, duration of surgery, rate of blood transfusion, and length of stay [16].

The American Society of Regional Anesthesia & Pain Medicine recommends the same anticoagulation precautions for deep nerve blocks, including LPB, as for neuraxial procedures for hematoma prevention [17]. LPB is used infrequently for analgesia following THA, possibly due to the general recommendation of chemoprophylaxis for deep vein thrombosis (DVT) post-THA [18], the availability of relatively safer, motor-sparing alternatives [3**]. Multiple serious complications are associated with LPB, including total spinal anesthesia, retroperitoneal hematoma, and renal puncture [19–21]. Although ultrasound guidance has enhanced safety of RA procedures [22, 23], the spread of local anesthetic to target nerve roots (T12-L4) after ultrasound-guided LPB remains unreliable [24], and clinical benefits likely do not outweigh the risks [3].

While LPB may provide adequate analgesia for THA, it may be inferior to suprainguinal fascia iliaca compartment block (SIFICB) in terms of readiness for discharge and block duration [25]. Like FNB and FICB, LPB also results in significant quadriceps weakness, delaying mobilization in the recovery period. Therefore, LPB may have an unfavorable impact on functional recovery compared to motor-sparing blocks such as QLB, PENG, and/or LFCN. In one RCT, Kelly et al. was unable to demonstrate noninferiority of the QLB over LPB but did find significantly greater quadriceps strength in PACU and improved mobilization following QLB [26].

Despite its decline in clinical use, LPB continues to be considered a valuable comparator in the academic evaluation of regional anesthesia (RA) techniques for hip analgesia. Its perceived ability to treat key nerves of the hip (LFCN, femoral, and obturator) in a single procedure makes LPB the standard against which many newer methods are compared for efficacy and effectiveness in treating hip pain [25, 27–29].

### Quadratus Lumborum Block

The quadratus lumborum block (QLB) is a fascial plane block which targets thoracolumbar nerve roots [30]. The use of QLB for postoperative analgesia of the hip gained attention when Uppal et al. published a meta-analysis in 2020 suggesting that QLB may be effective, broadly, for abdominal wall and hip surgery [31]. This meta-analysis was limited by heterogeneity in both patient characteristics and procedure type but offered QLB as a possible solution to the important clinical problem of optimizing analgesia following THA. At least 14 retrospective analyses and 21 randomized clinical trials evaluating the analgesic efficacy of QLB for patients undergoing THA were identified by our database search. They yield conflicting results regarding the impact on pain scores, time to ambulation, time of hospitalization, opioid consumption, and adverse effects such as PONV, and are likely underpowered to generate a meaningful level of certainty.

Five meta-analyses assessing QLB efficacy for THA have been considered for this review. Most studies included in these meta-analyses used the anterior approach to the QLB, but due to low power, subgroup analyses were not performed. Neither was a subgroup analysis of the surgical approach performed. Of the identified meta-analyses, Hussain et al., Hu et al., and Huda et al. improved heterogeneity of their analyses by including only RCTs and excluding RCTs with active comparators (for example, studies that compare QLB to FICB) [32–34]. All five RCTs synthesized by Huda et al. were also included by Hussain et al., and Hussain et al. had the largest study population (*n* = 1318). Hussain et al. reports statistically significant benefits of QLB for patients having THA including improved functional outcomes for patients who received general but not spinal anesthesia, decreased opioid related side effects such as PONV, decreased pain scores, and decreased opioid consumption. However, the reduction in opioid consumption and VAS failed to meet the predetermined minimal clinically important difference (PMCID) [35]. Hu et al. (*n* = 830) found the QLB had clinically significant reductions in VAS on mobilization at all time points, but at rest this reduction was not clinically significant. Opioid consumption was decreased in the QLB group but only demonstrated clinical significance at 48 h postoperatively [33]. Reduced opioid related side effects such as nausea and vomiting in the QLB group were reported by all three authors who evaluated this outcome. Patient satisfaction was also increased for all authors who reported this outcome [33, 34]. There is no PMCID defined for functional outcomes or opioid related side effects such as PONV, so these findings may not be clinically significant. All of these meta-analyses were underpowered to reliably assess differences between the two groups for pain scores and VAS. Brixel et al. discusses the need for 2,029 subjects in each group for sufficient power [36]. Therefore, the benefits of QLBs for elective THA may not outweigh the risks and costs. However, there is a low level of certainty, and results should be interpreted with caution.

In the meta-analyses which included active comparators comparing QLB to FICB and LPB) [37–39], QLB for THA did not consistently reduce pain scores or opioid consumption, which may suggest that QLB is not significantly superior to alternative PNBs for THA. Multiple RCTs suggest that QLBs probably do not provide superior analgesia compared to FNB, FICB, or LPB [26, 40–42]. However, compared to RA procedures involving the femoral nerve, QLBs offer the benefit of decreased rate of quadriceps weakness immediately after surgery [26, 39, 41, 43], thereby permitting earlier ambulation, which is an important benefit to consider following THA. Hummel et al. cautions against the anterior QLB, as they found higher rates of motor weakness compared to paravertebral block [44]. However, we note that it is highly unlikely to have motor weakness following QLB when performed properly; motor weakness only occurs upon violation of the psoas compartment, which mimics a lumbar plexus block. Moreover, Hummel et al. describe QLBs performed with bupivacaine hydrochloride 0.5% and the risk of motor blockade with high volume fascial plane blocks may be mitigated by alternate approaches and a lower (e.g. 0.25%) concentration of local anesthetic [45]. Therefore, when early ambulation is desirable, QLB may be an appropriate analgesic option when used either alone or alongside other motor-sparing RA techniques.

We identified several comparisons of PENG and QLB for THA. An RCT from Et et al. compared PENGs to QLBs to no blocks [46]. Both block groups had superior pain control compared to the no block groups, but the PENG and QLB groups showed similar postoperative quality of recovery. In another retrospective study, QLB outperformed PENG in PACU VAS but had significantly longer length of stay. Both QLB and PENG had superior analgesia to the control group (no block) [47]. There was no difference in opioid consumption among the block groups. In an RCT comparing PENG to QLB for THA, the PENG group reported significantly lower maximum pain scores in the PACU, 3-, and 6-hours following surgery. There was no significant difference in opioid consumption, length of hospitalization, or pain at one year following surgery [43]. In summary, while both PENG and QL provide analgesia for THA, it is not clear which, if either, is clinically superior.

QLB may provide additional analgesia when performed in combination with additional motor-sparing RA techniques such as PENG and LFCN blocks. Compared to no-block, QL & LFCN resulted in decreased mean VAS and opioid consumption following THA; only decreased opioid consumption met the PMCID [48]. One retrospective study of 210 patients who received LFCN, QLB, and periarticular injection for elective THA under spinal anesthesia showed decreased length of stay, decreased VAS in the PACU, and decreased postsurgical opioid consumptions compared to periarticular injection and spinal anesthesia alone [48]. Another retrospective cohort analysis (*n* = 16) [49], suggested that PENG plus QLB provided superior analgesia when compared to QLB alone for revision THA.

In conclusion, QLB alone may not provide clinically significant benefits for THA, especially when compared to other RA techniques. Multiple low-powered studies suggest that QLB has a role in motor-sparing regional anesthesia techniques for analgesia and expedited recovery following THA when combined with LFCN, PENG, and/or periarticular injection. However, it remains unclear if the benefits are sufficient to warrant the costs and risks of the procedure. While not uniformly clinically significant, QLB showed statistically significant benefits in all five meta-analyses and has been described for both rescue analgesia following THA [50] and in combination with FICB for surgical anesthesia [51]. Depending on the clinical context, QLB remains an important option in the perioperative management of THA. More high-powered studies are warranted to further define the role of routine QLB for THA.

### Pericapsular Nerve Group Block

The Pericapsular Nerve Group (PENG) block has emerged as a novel regional anesthetic technique that provides significant postoperative analgesia for those undergoing THA and traumatic hip fractures. Initially described in 2018, the block targets the articular branches of the femoral nerve, obturator nerve and accessory obturator nerves around the anterior hip capsule [52].

Local anesthetic is deposited within the myofascial plane of the psoas muscle and the superior pubic ramus, between the psoas tendon and ilium [53]. The PENG block can offer substantial pain relief without the extensive motor blockade associated with other regional techniques. Thus, it has emerged as a potentially useful motor-sparing block for THA [54].

By targeting the proximal articular branches that innervate the anterior hip capsule, the PENG block offers more complete analgesia to the hip joint compared to FNB or FICB. The PENG block has been reported to provide better analgesia and less quadriceps muscle weakness compared to fascia Iliaca blocks (both infrainguinal and SIFI approaches) for both THA and hip fractures [55, 56]. When compared with SIFICB, PENG block has resulted in better preservation of motor function [57]. Further, PENG block has been associated with faster discharge readiness, longer postoperative analgesic effect and improved quadriceps strength compared to femoral nerve block alone [58, 59].

Quality of recovery as measured by the Quality of Recovery-15 (QoR-15) questionnaire was consistently observed with the PENG block [60]. The QoR-15 is a validated patient outcome questionnaire used to assess postoperative recovery in terms of physical independence, pain, patient comfort, amongst several other measures. Scores for this questionnaire range from 0, an extremely poor quality of recovery, to 150, an excellent quality of recovery. Median QoR-15 scores were higher in those receiving PENG block (132 [116–138 IQR]) when compared with no block (103 [97–112 IQR]) [60]. However, when compared to femoral nerve block, there was no difference detected in terms of quality of recovery as measured by QoR-15 [59].

When comparing PENG block with periarticular local anesthetic infiltration in patients undergoing primary THA, comparable rates of quadriceps muscle weakness was observed [54]. While there were lower static pain scores at all measurement intervals, particularly in the first 24 h in the periarticular local anesthetic infiltration group, there was no difference in terms of time to first opioid request, cumulative morphine consumption, ability to perform physical therapy and length of stay [54]. Findings would suggest that the analgesic effect and functional recovery of PENG block was not superior to periarticular local anesthetic infiltration in THA [61].

Although the PENG block has significant motor sparing benefits, it can still cause transient quadriceps muscle weakness. Impairment in knee extension or hip adduction may stem from local anesthetic spread to the femoral nerve and obturator nerve respectively. Impairment of hip adduction may simply result from large volumes of injectate or if the needle tip is positioned medially along the iliopubic eminence resulting in obturator motor blockade [57]. Aliste et al. in a randomized trial comparing PENG with SIFICB for THA found that 95% of patients receiving a PENG block had no motor block with knee extension at 24 h. This is further illustrated by Lin et al. in 2021, who found 90% of patients receiving PENG block had intact motor function on postop day 1 versus 50% of patients who received femoral nerve block [58].

In summary, the PENG block can be an option in achieving effective motor-sparing postoperative analgesia in THA. Potential benefits may include reduced postoperative opioid consumption and improved quality of recovery. However, potential lower extremity weakness and limited evidence of efficacy compared to periarticular injection remain as considerations in its use.

### Femoral Nerve and Fascia Iliaca Blocks

The femoral nerve block has been used for analgesia after both total hip arthroplasty (THA) and Total Knee Arthroplasty (TKA). The rationale for its use in THA is derived from the osteotomes of the hip joint; these are primarily innervated by the femoral, obturator, and sciatic nerves. Thus, a femoral nerve block can provide partial analgesia to the hip capsule. For example, recently Aoyama et al. compared the use of a quadratus lumborum catheter and femoral nerve catheter and reported superior analgesia in the femoral nerve catheter group [40]. However, the use of femoral blockade for THA has classically been limited by (1) its limited application in THA incisions along the lateral portion of the thigh, and (2) the risk of motor blockade, particularly its potential negative influence on patient fall risk [62]. Thus, use of femoral blockade for THA remains not without risk [63, 64].

The fascia iliaca block, first described in 1989, is similar to the femoral nerve block in anesthetic distribution but also involves the lateral femoral cutaneous nerve (LFCN) which arises from the lumbar plexus and provides sensory innervation to the lateral thigh. This additional coverage may be beneficial in posterolateral THA. While some studies report effective pain relief with limited motor blockade (likely secondary to a more dilute local anesthetic injectate), at least one randomized control trial did not report a difference in pain scores compared to placebo [65]. Concerningly, in this study, patients in the fascia iliaca group had a 22% incidence of quadriceps muscle weakness [63, 66, 67].

A variation of the classical femoral nerve block and fascia iliaca block, the suprainguinal fascia iliaca block (SIFICB), has also been described in the literature. This block aims to target the femoral, lateral femoral cutaneous and obturator nerves. While this block has resulted in reduced pain scores and opioid use compared to placebo, when compared to periarticular infiltration no statistical difference in pain scores or opioid consumption has been observed [68]. Additionally, patients who receive SIFICB may have a significant increase in risk of motor blockade up to six hours after injection [62].

In summary, while a femoral nerve block and its variations can provide some analgesia in THA, the use of these non-motor-sparing blocks carries a certain risk of muscle weakness which can hinder ambulation or increase the risk of patient falls. Therefore, their use after hip arthroplasty, which requires prompt postoperative ambulation, is limited. Furthermore, lack of analgesic advantages compared to periarticular injection in elective primary THA further limits clinical applicability.

## Conclusions

**Fig. 1:**
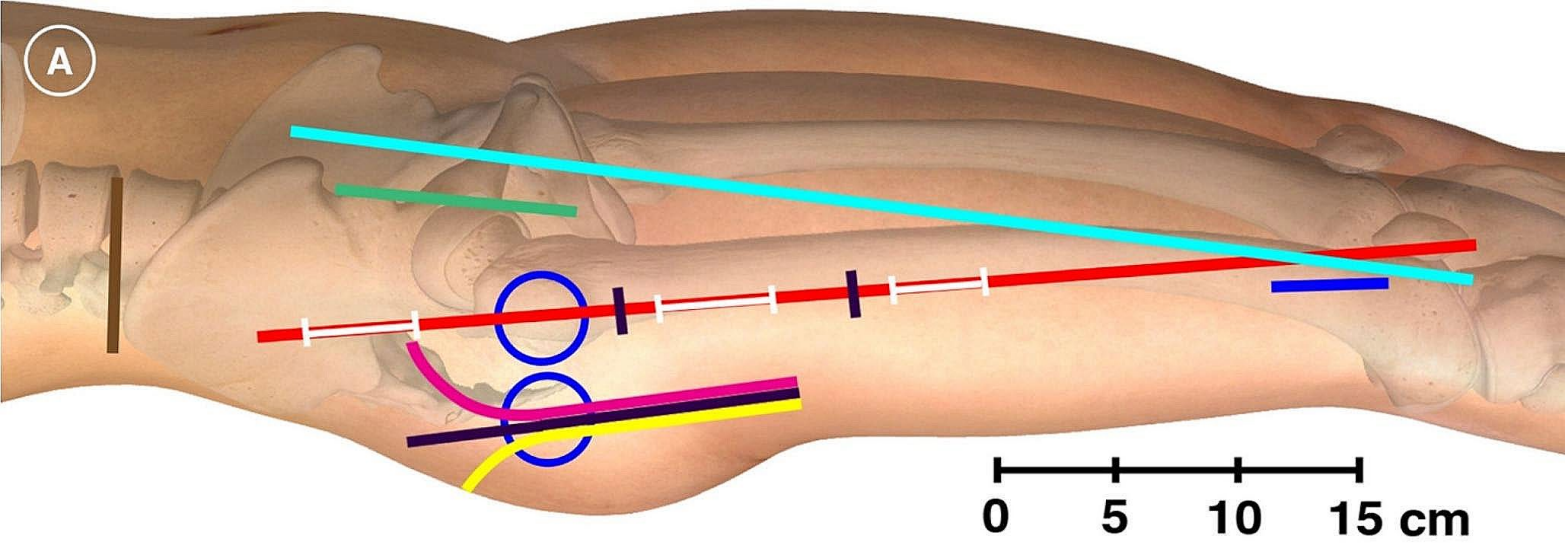
Cutaneous anesthesia of hip surgery incisions with iliohypogastric and subcostal nerve blockade: A randomized trial. Graphical depiction of the reference and incision lines marked at the beginning of the trial with an ultraviolet marker. Anterior blue circle, surface projection of the greater trochanter with the subject in the supine position and 20° internal rotation; posterior blue circle, surface projection of the greater trochanter with the subject in the lateral position; red line, GT-P‐line (straight line intersecting the greater trochanter and the lateral edge of the patella); cyan line, ASIS‐GST‐line (straight line intersecting the ASIS and Gerdy’s tubercle); brown line, straight line through the most cranial point of the iliac crest (IC‐line); green line, anterior incision; magenta line, anterolateral incision; black line, direct lateral incision; yellow line, posterior incision; proximal white line on the red line, incision for entry point of intramedullary (IM) nail; intermediate white line on the red line, incision for proximal locking of an IM nail; distal white line on the red line, incision for distal locking screws of a short IM nail; blue line, incision for distal locking screws of a long IM nail; the red line segment between the two black markings, incision for sliding hip screw (SHS) or parallel implants (parallel screws or hook‐pins). The figure is a modified excerpt from Essential Anatomy 5, with permission from 3D4Medical (www.3d4medical.com). This image has been used with permission from the publisher. It has been modified by exclude the B part of the original image.

Overall, recent evidence for the use of regional anesthesia for THA is mixed. The FNB, PENG, and FICB techniques have received the most attention and provide respectable analgesia while others like the ESP and PVB have relatively less evidence supporting their use. Further, none of these approaches address all cutaneous, osteotomal, and myotomal transgressions for any of the described surgical approaches. Figure 1A (used with permission) depicts many of the common surgical approaches for THA and femur fracture surgery.  1[69]. Given the heterogeneity of evidence, the lack of consensus PNB technique, and the numerous nerves that contribute to pain in THA, surgeons will at times opt for local infiltration analgesia (LIA), where the surgical team infiltrates the hip capsule and dissected layers with local anesthetic with or without various adjuncts like epinephrine, ketorolac, or dexamethasone [70]. This may be performed in lieu of PNB or may be combined with various PNBs. This combination approach may further improve postoperative pain [71].

It is important to note that the International Consensus on Anesthesia-Related Outcomes after Surgery (ICAROS) Group, in a meta-analysis of the current literature in 2021, provide a strong argument for the use of PNBs. Based on a large, multi-database review, the group analyzed the effect of nerve blocks on various outcomes after surgery, such as cognitive dysfunction, respiratory failure, cardiac complications, surgical site infections, thromboembolism, and blood transfusion in over one million patients [2]. They found decreased odds ratios in all the above-mentioned risks and concluded that with moderate to strong amount of evidence, that all patients getting THA (and TKA for that matter), should receive PNBs. Some limitations of the ICAROS group recommendations include the lack of PENG blocks in the analysis, though this may have only strengthened their association, as the RCTs associated with the PENG block have shown improved outcomes. It should be noted that a weakness of their study is that it did not *only* include RCTs, but observational studies as well.

This important study returns us to the question of whether the patient should receive a block for THA, and the evidence suggests the answer is yes. The specific block chosen should be influenced by provider comfort, surgical approach, patient anatomy, and postoperative goals. There exists evidence that some PNBs may not be better than LIA or may be improved by addition of LIA to improve postoperative pain, and further study is needed in this context [71]. Further, there are relatively few comparative studies comparing PNB techniques in a head-to-head fashion.

In conclusion, THA is a common and growing surgical technique that continues to be refined for outpatient surgery. The recent improvements in combinations of appropriate surgical technique, multimodal analgesia, surgical local infiltration, and a patient-tailored peripheral nerve block will improve patient outcomes now and in the future.

## Data Availability

No datasets were generated or analysed during the current study.
